# Non-invasive estimation of hemoglobin, bilirubin and oxygen saturation of neonates simultaneously using whole optical spectrum analysis at point of care

**DOI:** 10.1038/s41598-023-29041-w

**Published:** 2023-02-09

**Authors:** Amrita Banerjee, Neha Bhattacharyya, Ria Ghosh, Soumendra Singh, Aniruddha Adhikari, Susmita Mondal, Lopamudra Roy, Annie Bajaj, Nilanjana Ghosh, Aman Bhushan, Mahasweta Goswami, Ahmed S. A. Ahmed, Ziad Moussa, Pulak Mondal, Subhadipta Mukhopadhyay, Debasis Bhattacharyya, Arpita Chattopadhyay, Saleh A. Ahmed, Asim Kumar Mallick, Samir Kumar Pal

**Affiliations:** 1 Department of Physics, Jadavpur University, 188, Raja S.C. Mallick Rd, Kolkata, 700032 India; 2 Technical Research Centre, S. N. Bose National Centre for Basic Sciences, Block JD, Sector III, Salt Lake, Kolkata, West Bengal 700106 India; 3 Department of Paediatric Medicine, Nil RatanSircar Medical College & Hospital, 138, AJC Bose Road, Sealdah, Raja Bazar, Kolkata, 700014 India; 4 Department of Radio Physics and Electronics, University of Calcutta, 92, Acharya Prafulla Chandra Rd, Machuabazar, Kolkata, 700009 India; 5 Department of Chemical and Biological Sciences, S. N. Bose National Centre for Basic Sciences, Block JD, Sector 3, Salt Lake, Kolkata, 700106 India; 6 Neo Care Inc, 27, Parker St, Dartmouth, NS B2Y 2W1 Canada; 7 Electrical and Computer Engineering Department, Dalhousie University, 6299 South St, Halifax, NS B3H 4R2, Halifax, Canada; 8 Chemical and Biomolecular Engineering, University of California, Los Angeles, CA 90095 USA; 9 Department of Applied Optics and Photonics, JD-2, Sector-III, Salt Lake, Kolkata, West Bengal 700 106 India; 10 Department of Biotechnology, Thapar Institute of Engineering and Technology, Bhadson Road, Patiala, Punjab 147004 India; 11 Faculty of Medicine, Assiut University, Assiut, 71516 Egypt; 12 Department of Chemistry, College of Science, United Arab Emirates University, Al Ain, P.O. Box 15551, Abu Dhabi, United Arab Emirates; 13 Department of Gynecology & Obstetrics, Nil Ratan Sircar Medical College & Hospital, 138, AJC Bose Road, Sealdah, Raja Bazar, Kolkata, 700014 India; 14 Department of Basic Science and Humanities, Techno International, Kolkata, 700156 India; 15 Department of Physics, Sister Nivedita University, Kolkata, India; 16 Department of Chemistry, Faculty of Applied Science, Umm Al-Qura University, Makkah, 21955 Saudi Arabia

**Keywords:** Biochemistry, Biological techniques, Biophysics, Chemical biology, Microbiology, Medical research, Analytical chemistry, Biochemistry, Chemical biology, Medicinal chemistry

## Abstract

The study was aimed to evaluate the performance of a newly developed spectroscopy-based non-invasive and noncontact device (SAMIRA) for the simultaneous measurement of hemoglobin, bilirubin and oxygen saturation as an alternative to the invasive biochemical method of blood sampling. The accuracy of the device was assessed in 4318 neonates having incidences of either anemia, jaundice, or hypoxia. Transcutaneous bilirubin, hemoglobin and blood saturation values were obtained by the newly developed instrument which was corroborated with the biochemical blood tests by expert clinicians. The instrument is trained using Artificial Neural Network Analysis to increase the acceptability of the data. The artificial intelligence incorporated within the instrument determines the disease condition of the neonate. The Pearson’s correlation coefficient, r was found to be 0.987 for hemoglobin estimation and 0.988 for bilirubin and blood gas saturation respectively. The bias and the limits of agreement for the measurement of all the three parameters were within the clinically acceptance limit.

## Introduction

Neonatal jaundice, anemia and hypoxia are the most common health issues encountered by newborns globally and constitute a major percentage of infant mortality. The prevalence of neonatal jaundice, anemia and hypoxia is quoted to be between 50 and 60% among healthy term neonates^1–3^. According to the recent reports of World Health Organization, neonatal jaundice affects one in every two infants globally. One of the major reasons for pathologic hyperbilirubinemia is the excessive production of bilirubin, a byproduct of hemoglobin breakdown, and the impaired ability of the newborn to excrete it^4^. Among the reported cases of neonatal hyperbilirubinemia, about 15% of the neonates suffer from persistent jaundice that lasts for about 14 to 21 days^5^. All these infants suffering from persistent jaundice have significantly decreased hemoglobin levels^6^ and elevated bilirubin concentration in blood due to the increased bilirubin production from hemolysis resulting in a simultaneous pathologic condition of jaundice and anemia among neonates^4,6^. According to the American Academy of Pediatrics (AAP), the incidence of neonatal hyperbilirubinemia is increased among infants having risk factors like, ABO incompatibility, Rh incompatibility, birth asphyxia, etc.^7^. It has been reported that the occurrence of neonatal jaundice is more likely among neonates suffering from birth asphyxia, than neonates without birth asphyxia^8–11^ due to the lack of oxygen supply to the liver which results in hypoxic damage followed by the bilirubin conjugation ability of the liver, which ultimately results into jaundice^8^. Additionally, perinatal asphyxia and hypoxic-ischemic encephalopathy can lead to the disruption of the blood–brain barrier, allowing the free entry of unconjugated bilirubin to the neurons resulting in acute bilirubin encephalopathy^8^. In addition to this, dysregulation of blood flow to the lungs due to hemolysis can also cause an imbalance in the ventilation and perfusion ratio thereby, resulting in a hypoxic condition^12^ in the neonates^13^. Thus, simultaneous monitoring of bilirubin, hemoglobin and oxygen saturation levels in newborns is essential to ensure appropriate management. The contemporary method of measurement of serum bilirubin concentrations (TSB) hemoglobin levels (Hb) and arterial blood gas (for measurement of blood oxygen saturation) involves painful blood sampling^14–17^ which suffers from multiple long term consequences like infection at the sampling site, osteomyelitis (though in rare cases), blood loss, etc.^15,18,19^. Although, non-invasive methods (BiliCheck™^20^, JM-105™^21^, Rad 57™^22^ NBM-200, etc.)^23^ have been established as alternatives to repeated blood samplings for TSB and Hb measurement^24,25^ however, they suffer from certain inherent limitations that restrict their usage in widespread hospital settings^24,26,27^. Particularly, the accuracy of these non-invasive devices vary across races and have been found to be less accurate in Asian, Hispanic and African populations (having dark skin tone)^28^. On the other hand, the available transcutaneous pulse oximeters are the state-of-the art technology for the continuous estimation of oxygen saturation among neonates. Although, the pulse oximeters are correlated with the blood oxygen saturation and has been used in the hospital setting for more than a decade now, they suffer from certain limitations which may be improved to increase their acceptability. The pulse oximeters are vulnerable to the motion of the subject and, gives erroneous results by interpreting the motion of the infant as a pulse signal^29^. Moreover, these pulse oximeters overestimate the arterial oxygen saturation (SpO_2_) at less than 90% saturation thereby, limiting their usage in infants suffering from heart disease^30,31^. Therefore, monitoring of bilirubin, hemoglobin and SpO_2_ values is needed at the bedside of the neonate suffering from either jaundice, anemia or hypoxia for the proper management.

In this study, we aim to develop a non-invasive point of care device (i.e. SAMIRA, Spectrum Assisted Medical Inoffensive Radiation Application) for the simultaneous determination of hemoglobin, bilirubin and oxygen saturation in neonates. The newly developed instrument utilizes an algorithm to quantify three blood parameters from a single discrete measurement. The simultaneous measurement of bilirubin, hemoglobin and oxygen saturation from neonates in a non-invasive manner from a single measurement for their proper management is the motivation of the current work. As the device collects data from the blood vessels of the distal subungual arcade and the superficial arcade area discarding the influence of melanin or skin colour, it is hypothesized to give accurate transcutaneous measurement values of TSB, Hb and SpO_2_. Additional incorporation of machine learning and artificial intelligence has improved the data accuracy of the proposed device. By virtue of the machine learning algorithm the developed device is capable of locking a data which is 98% accurate. Studies on a huge number of neonatal populations helped in exact incident light dosage determination, data acquisition time optimization etc., making the device highly precise and accurate for point of care settings. Till date, to the best of our knowledge no device has been developed that can monitor these three blood parameters at the same time in a non-invasive way.

## Materials and methods

### Hardware

**Figure 1:**
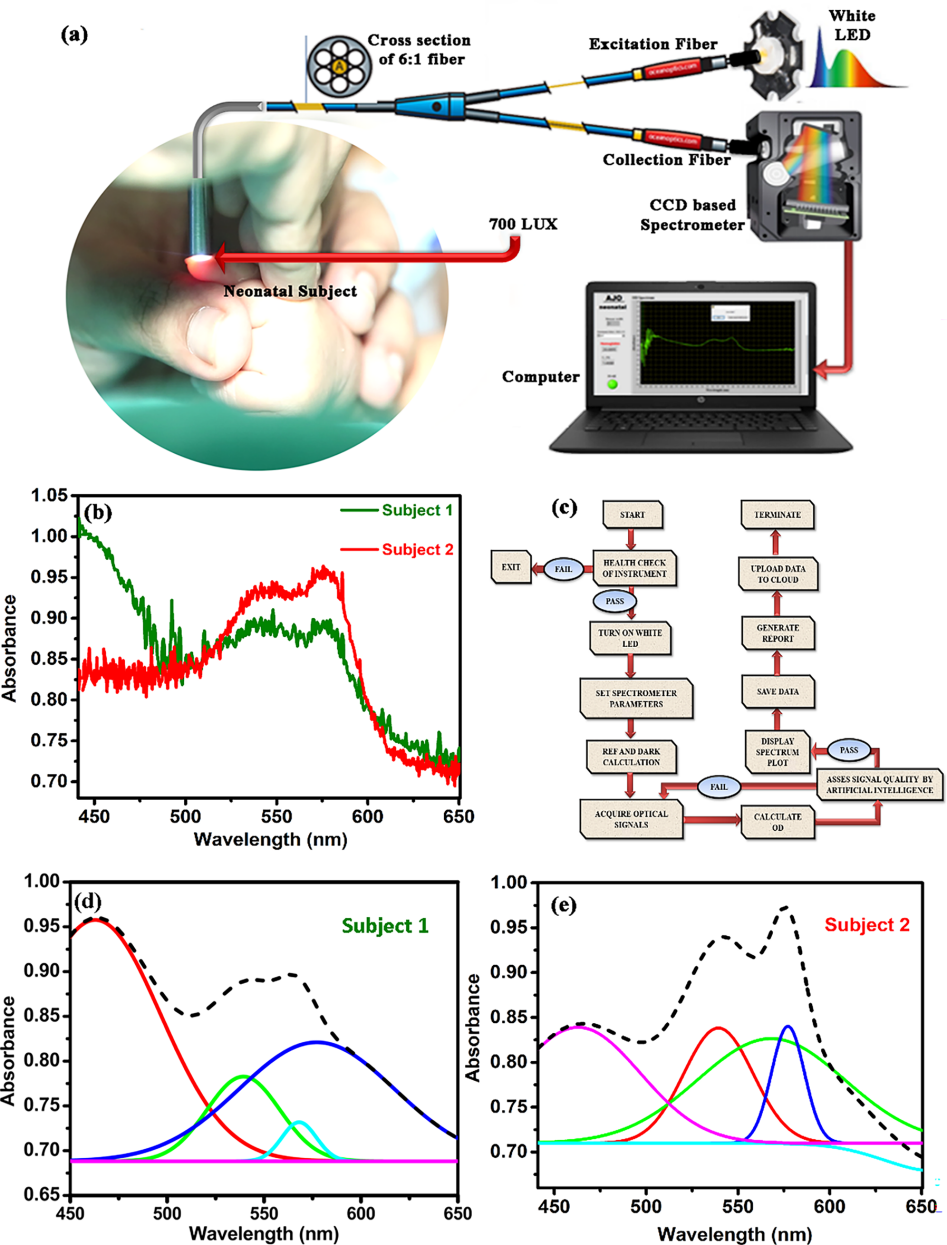
Design of the developed prototype with the acquired data and the de-convoluted spectrum. (**a**) The instrument consists of a LED source, a 6:1 optical fibre, a CCD based spectrophotometer and an integrated electronic module (See text for details). (**b**) The acquired spectra from two subjects. Subject 1 is diseased with TSB value 29.8 mg/dL; Hb value 10.2 gm/dL; and SpO_2_ value of 92%. Subject 2 is from a normal infant with TSB value 2.4 mg/dL; Hb value 19.1 gm/dL and SpO_2_ value of 95%. (**c**) Work flow of the instrument (**d**) De-convoluted peaks from subject 1 along with the cumulative fit (**e**) De-convoluted peaks from subject 2 along with the cumulative fit (see text).

The instrumental set up as shown in Fig. 1a has been designed based on the principle of diffused reflectance spectroscopy. The diffused reflectance spectroscopy (DRS) technique is based on the amount of light collected from the tissue layers after penetration of the incident light^32–34^. The retro-reflected light thus contains information about the tissue microstructure and the biomolecular content. The thumb nail plate of neonatal subjects is illuminated with light from a white Light Emitting Diode (LED) source (3 W, 400–700 nm, 700 LUX, 4.78 mW optical power) and the diffused optical signal in the visible range was collected in a CCD based spectrograph (STS-VIS, manufactured by Ocean Optics, Florida) with wavelength resolution of 0.47 nm. A lab grade 6:1 diffuse reflectance fiber optic probe manufactured by Ocean Optics, Florida was used to transmit the light from source to subject nail by the 6 peripheral excitation fibers of the probe and receive the response signal by the central collection fiber by holding the probe tip at perpendicular position with the nail. The obtained spectral response in the spectrometer is then transferred to a computer through USB connection for processing in our developed graphical user interface in LabVIEW (National Instruments) platform. Subsequent data acquisition, analysis and result generation are taken care of by the custom-made software. Proper cooling arrangements (5 V DC fans, 0.2 Amp) for dissipation of excess heat generated by components have also been incorporated in the instrument by virtue of which the device performance remain unaltered under varying temperature conditions. In the customized power supply module driven device, artificial intelligence is also implemented by the software automatically to filter out reliable spectrum after data acquisition by guiding the user to take more data unless appropriate accuracy level is achieved. Figure 1b reveals the distinct difference in the spectral signature of blood for a control neonate (TSB of 2.4 mg/dL, Hb of 19.1 gm/dL and arterial blood gas of 95%) in comparison to a sick neonate (TSB of 29.8 mg/dL, Hb of 10.2 gm/dL and arterial blood gas of 92%).

A graphic user interface was developed using LABVIEW software (National Instruments) for data acquisition, data analysis and generation of subsequent results^35,36^. The algorithm for the work flow of the device is shown in Fig. 1c. The whole blood spectrum obtained from the neonate’s nail bed was de-convoluted to obtain five independent signals at five wavelengths (462.92 nm, 539.34 nm, 568.09 nm, 577.2 nm and 620 nm). A comparative spectral response between a jaundiced and anemic infant (Subject 1) and a normal (Subject 2) and their de-convoluted spectra is shown in Fig. 1d,e.

### Machine learning algorithm

The present study uses a sophisticated machine learning (ML) algorithm known as Artificial Neural Networks (ANN) to analyze the vast amount of data-set collected during the study. The main objective is to introduce artificial intelligence (AI) framework through machine learning (ML) techniques, which are dedicated algorithms to train the software to learn from data^37^.

The ANN algorithm tries to imitate the network of a human brain by learning tasks and solving problems^38^. The input layers and output layers of the network are connected by single or multiple hidden layers and interconnecting nodes with variable ‘weight factor’^39^.

Utmost care was taken for spectrum data acquisition through the developed prototype. Figure 1c illustrates the simple sequential program flow diagram of the software for accurate assessment of blood hemoglobin, bilirubin and oxygen saturation levels in neonates. After getting powered up, health check-up and initialization of the instrument takes place. If there is any discrepancy, the device auto-corrects different conditions and restarts automatically, followed by a pop-up window asking for patient details including name, age, sex, medical conditions etc. to be saved along with data in individual folders. The software next guides to store ‘reference’ and ‘dark’ spectra one time for a particular ambient condition. The dark spectrum was acquired in the presence of ambient light by turning off the source LED. The effect of light scattering from the nail bed was taken into account by recording the reference spectrum. The reference spectrum was collected using a standard scatterer (WS-1 Reflectance Standards, Ocean Optics) with a spot size of 0.7 cm in diameter. The distance between the probe tip and the scatterer was maintained perpendicularly at a distance of ~ 1.5 cm, such that the maximum light was scattered. The pre-acquired dark spectrum and reference spectrum, (which were acquired each day before starting the data collection) were read from the preloaded file location for spectrum processing. It is to be noted that the dark and reference spectra were acquired each day before starting the data collection to avoid the potential effects of variation in ambient light. In case of a change in the measurement location both spectra were re-acquired. The integration time of the spectrometer was kept fixed at 3000 ms and the boxcar width (Smoothening factor/running average) at 2 in this entire study for maintaining a proper signal to noise ratio (S/N) of the spectra. The average time to acquire reliable data using the device is approximately 30 s, whereas the time required to communicate the acquired data to the cloud is nearly 1 min.

**Figure 2:**
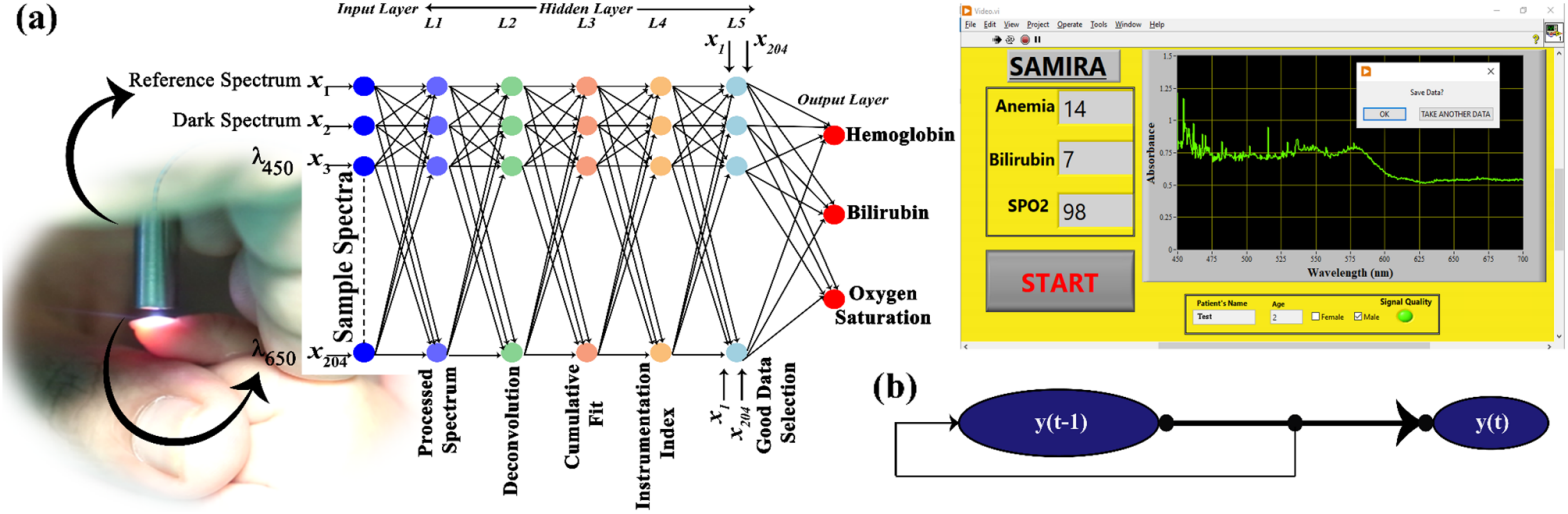
Machine Learning Algorithm (**a**) Schematic representation of ANN used in the proposed device for the detection of Hemoglobin, Bilirubin and Oxygen Saturation (**b**) Time dependent continuous series flow diagram to estimate the blood parameters in regular time intervals (see text).

For data processing mechanism, the following structure describes the various layers of ANN used for the present study (Fig. 2).

#### Input layer

The dark, reference, and sample spectra containing the absorbance values from 450 to 650 nm are the primary elements used by the input layer.

#### Hidden layer L1

This layer generates the processed spectrum data using the following equation

1 $$\mathrm{Processed \; spectrum}= -{\mathrm{log}}_{10}(\frac{\mathrm{Sample \; Spectrum}-\mathrm{Dark \; Spectrum}}{\mathrm{Reference \; Spectrum}-\mathrm{Dark \; Spectrum}})$$

The recording of the dark and reference spectra and the processing of the acquired signal in accordance to Eq. (1), compensates the impact of light scattering from the neonate’s nail bed. A training statement was also introduced in this layer which limits the amplitude of the absorbance range within 0.5 and 0.6 at 620 nm. This was maintained by the following instruction: 0.5 ≤ Abs _620 nm_ ≥ 0.6. This condition was given in order to maintain the spot size to be 0.7 cm and the distance between the probe tip and nail bed to be 0.6 cm.

#### Hidden layer L2

Layer for de-convolution of the processed spectrum (raw signals) and assessing the residuals (amount of scattered data from the fitted line) of the fit parameters to be acceptable by the system. The whole blood spectrum collected from the neonates’ nail bed was de-convoluted into five Gaussian functions (Fig. 1d,e). The equation for each Gaussian function (y) is as follows:

2 $${\text{y}} = {\text{y}}_{0} + {\text{A exp}}\;\left( { - 0.{5}\;\left( {\left( {{\text{x}} - {\text{x}}_{{\text{c}}} } \right)/{\text{w}}} \right)^{{2}} } \right)$$

where y_0_ is the offset, A is the amplitude of the Gaussian curve, w is the full width by half maxima (FWHM) and x_c_ is the peak wavelength of the Gaussian curve. Each of the five Gaussian curves has a fixed peak wavelengths at 462.92 nm, 539.34 nm, 568.09 nm, 577.2 nm and 620 nm. The peak wavelengths were chosen on the basis of the pattern of absorption of oxygenated hemoglobin, de-oxygenated hemoglobin and of bilirubin.

This layer is also responsible for generation of membership functions, where the peak wavelength of 462.92 nm corresponds to the absorption of bilirubin, 539.34 nm and 577.2 nm corresponds to the Q bands of oxy-hemoglobin and that of 568.09 nm corresponds to the de-oxygenated peak of hemoglobin. The additional peak at 620 nm has been considered to correct the baseline and scattering contributions from the acquired spectrum.

#### Hidden layer L3

In this layer a cumulative fit of the data was procured by adding each of the independent five Gaussian curves as follows.

3 $${\text{y}} = {\text{y}}_{0} + {\text{A}}_{{1}} {\text{exp}}\;\left( { - 0.{5}\;\left( {\left( {{\text{x}} - {\text{x}}_{{{\text{c1}}}} } \right)/{\text{w}}_{{1}} } \right)^{{2}} } \right) + {\text{A}}_{{2}} {\text{exp}}\;\left( { - 0.{5 }\left( {\left( {{\text{x}} - {\text{x}}_{{{\text{c2}}}} } \right)/{\text{w}}_{{2}} } \right)^{{2}} } \right) + {\text{A}}_{{3}} {\text{exp}}\;\left( { - 0.{5}\;\left( {\left( {{\text{x}} - {\text{x}}_{{{\text{c3}}}} } \right)/{\text{w}}_{{3}} } \right)^{{2}} } \right) + {\text{A}}_{{4}} {\text{exp}}\;\left( { - 0.{5 }\left( {\left( {{\text{x}} - {\text{x}}_{{{\text{c4}}}} } \right)/{\text{w}}_{{4}} } \right)^{{2}} } \right) + {\text{A}}_{{5}} {\text{exp}}\;\left( { - 0.{5 }\left( {\left( {{\text{x}} - {\text{x}}_{{{\text{c5}}}} } \right)/{\text{w}}_{{5}} } \right)^{{2}} } \right)$$

where A_1_ A_2_, A_3_, A_4_ and A_5_ is the amplitude of the Gaussian curves with peak wavelengths at 462.92 nm, 539.34 nm, 577.2 nm, 568.09 nm, and 620 nm respectively, as mentioned earlier. In this layer, the area under each of the Gaussian curves is calculated using the Trapezoidal Rule, using the cumulative fitted equation. Assuming f(x) to be continuous over [a,b], the area under each of the Gaussian curve has been calculated using,

4 $$\underset{n\to \infty }{\mathrm{lim}}{T}_{n}= {\int }_{a}^{b}f\left(x\right) dx$$

#### Hidden layer L4

The instrumentation indices for the parameters were identified in this layer using the area under the five Gaussian curves and appropriate weightage was given to each one of them. It was noted that, summation of the area under the curves (AUC) corresponding to the wavelength of 539.34 nm and 577.2 nm (AUC_539.34_ + AUC_577.2_) provide an estimation of haemoglobin as the absorption band at 539 nm and 577 nm of the blood spectrum corresponds to the absorption of oxygenated hemoglobin and the trough at 568 nm corresponds to the de-oxygenated hemoglobin^40,41^. Similarly, bilirubin and oxygen saturation were calculated by measuring the AUC_462.92_ (as 462 is the characteristic peak of bilirubin^42^*)* and AUC_577.2_/AUC_568.09_ respectively. The amplitude of the Gaussians changed in relation to the amount of the biomolecule (bilirubin and hemoglobin) present. The change in the AUCs was mapped with the gold standard to obtain the calibration curve.

#### Hidden layer L5

**Figure 3:**
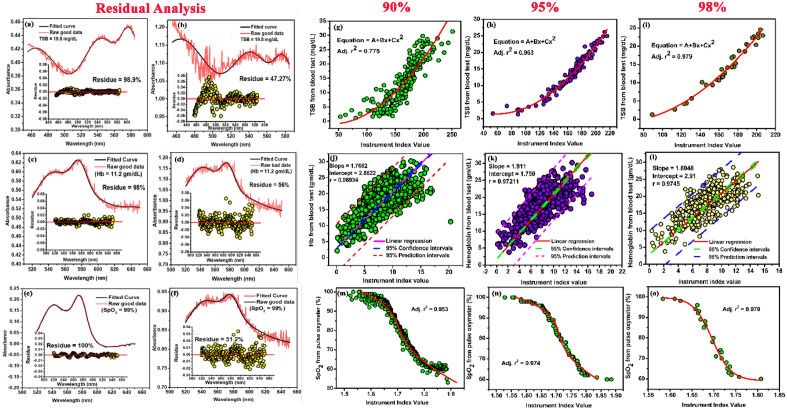
Training sets of the device using the self-devised algorithm (**a**,**c**,**e**) Raw and fitted data with the scattered residual points between ± 0.02% from the cumulative fitted data, used for the estimation of TSB (19.8 mg/dL), Hb (11.2 gm/dL) and SpO_2_ (99%). (**b**,**d**,**f**) Raw and fitted data with the scattered residual points beyond ± 0.02% from the cumulative fitted data, discarded by the algorithm for the estimation of TSB (19.8 mg/dL), Hb (11.2 gm/dL )and SpO_2_ (99%). (**g**–**i**) The calibration curve between the instrument index values acquired from the device with the obtained TSB values from blood test at the three partitions (see text) in 229 neonates respectively. The calibration curve shows a polynomial nature with the TSB values and the instrument index function. (**j**–**l**) The calibration curve between the instrument index values acquired from the device with the obtained Hb values from blood test at the three partitions (see text) in 1072 neonates respectively. The calibration curve shows a linear dependency with the Hb values and the instrument index function. (**m**–**o**) The calibration curve between the instrument index values acquired from the device with the obtained ABG values from biochemical test at the three partitions (see text) in 483 neonates respectively. The calibration curve shows a polynomial nature with the ABG values and the instrument index function.

In this layer the residual number of points of the fitted data from the acquired spectrum was analysed. The amount of the scattered data from the acquired signal with respect to the pre specified parameters given in hidden layer L3 is analysed in this layer. For the acceptability of the data, 98% of the residual data points necessarily must be within the range of ± 0.02% from the cumulative fitted curve. On the contrary, the scattered data points beyond ± 0.02% will be rejected. A comparison between the accepted data and the rejected data is shown in Fig. 3a–f.

Initially, the machine was trained to accept the spectra, when 90% of the residual data points of the fitted data were within the range of ± 0.02%. The Adjusted r^2^ for quantification of hemoglobin, bilirubin and oxygen saturation (Table 1) was found to be 0.77, 0.96 and 0.95 respectively for this training data set (Fig. 3g). Thus, a library containing the hemoglobin, bilirubin and oxygen saturation values of 483 neonates was prepared. The prototype-specific instrument indices were clinically validated using a regression analysis (Fig. 3)^43,44^. The minimization problem was utilized to minimize the error between the predicted value and the actual value using the following equations

5 $$Minimize\frac{1}{n}{\sum }_{i=0}^{n}{({predi}_{i}-{y}_{i})}^{2}$$

6 $$J=\frac{1}{n}{\sum }_{i=0}^{n}{({predi}_{i}-{y}_{i})}^{2}$$

where, J is the minimization function. The difference between the predicted values and the acquired values measures the error difference.

**Table 1:** Statistical parameters defining the correlation of the three partitions of the calibration dataset (see text for details) with the three blood parameters.

Amount of scattered data from the cumulative fitted curve	Statistical test	Parameters	Hb measurement	TSB measurement	SpO_2_ measurement
90%	Linear regression analysis	Regression Coefficient (r)	0.77	0.74	0.70
		P value	< 0.0001	< 0.0001	< 0.0001
		Slope	0.9117	0.852	0.961
		Intercept	1.1749	2.564	2.375
95%	Linear regression analysis	Regression Coefficient (r)	0.87	0.84	0.88
		P value	< 0.0001	< 0.0001	< 0.0001
		Slope	0.917	0.94	1.0294
		Intercept	1.211	0.844	2.098
98%	Linear regression analysis	Regression Coefficient (r)	0.98	0.98	0.98
		P value	< 0.0001	< 0.0001	< 0.0001
		Slope	0.9548	1.006	0.977
		Intercept	0.6686	0.372	1.775

The Mean Squared Error (MSE) function over all the data points, has been computed by squaring the error difference and summed over all data points and divided that value by the total number of data points.

To reduce the error the machine was trained to accept the spectra, when 95% of the residual data points of the fitted data were within the range of ± 0.02%. The Adjusted r^2^ for quantification of hemoglobin, bilirubin and oxygen saturation was found to be 0.96, 0.97 and 0.95 respectively for this training data set (Fig. 3). However, the standard deviation was ± 5.4 for the estimation of the three blood parameters. Finally, the machine was trained to accept the spectra, when 98% of the residual data points of the fitted data were within the range of ± 0.02%. The Adjusted r^2^ for quantification of hemoglobin, bilirubin and oxygen saturation was found to be 0.96, 0.97 and 0.95 respectively for this training data set (Fig. 3). The hidden layer L5 basically identifies good data and bad data, through the error calculation. Figure 3a–f shows clearly, how the good data is remarkably different from bad data in terms of the amount of scattered data from the cumulative fitted line.

The processed data in a data library are deposited locally as well as in the cloud-storage. Provision of a dynamic calibration library strengthens the overall algorithm of the device by iterative method.

This layer is responsible for decision making protocol as well. Identification of a good data leads to output layer, where blood report would be generated, whereas, for bad data, back-propagation technique would be adopted.

#### Output layer

Output layer estimated the hemoglobin, bilirubin and oxygen saturation of the neonates and displayed them. Consequently, it also displayed whether the neonate is suffering from, either anemia, jaundice and hypoxia. The IOT enabled device enables its user to send blood reports via email and SMS. If anemia is detected, the online report may be dispatched to the doctor or patient party to expedite the treatment procedure. Finally, a dialog box appears to ensure that whether the job is to be terminated or repeated. The easy and user- friendly operating software interface makes the device to be handled by any layman without any prior medical or instrumentation knowledge.

The developed prototype can detect the blood parameters in equal time intervals and is suitable for time series monitoring governed by the following equation:

7 $${\text{y}}\;\left( {\text{t}} \right) = {\text{f}}\;\left( {{\text{y}}\;\left( {{\text{t}} - {1}} \right) \ldots \ldots {\text{y}}\;\left( {{\text{t}} - {\text{d}}} \right)} \right)$$

For the neonates undergoing phototherapy, the equal interval time series analysis would be compared with the Bhutani nomogram^7^, to detect the risk level associated with infant's hours of age and serum bilirubin concentration. For haemolytic anaemic patients and neonates suffering from heart diseases or hypoxia, time dependent data monitoring of haemoglobin and oxygen saturation will be crucial for treatment management and to determine the future courses of therapeutic action.

### Experimental protocol

The experimental protocol consisted of the data collection using the in-house device (SAMIRA), parameters were calculated from the data. The measurements using the device was recorded at the same time blood was collected from neonate. The blood was sent for analysis to estimate the TSB, Hb and SpO_2_, which was corroborated by a statistician blinded to the entire study. A single measurement was taken from the neonate’s thumb nail bed to estimate three blood parameters simultaneously.

### Study settings

This was a prospective observational study conducted over 25 months starting from January 2017 at the Department of Pediatric Medicine, Nil Ratan Sircar Medical College and Hospitals (NRSMH, a Govt. aided tertiary hospital), Kolkata, India.

### Sample size estimation

The sample size was estimated using the Everald’s equation for power calculation in diagnostics tests^45^. Assuming the expected lowest sensitivity (SN) to be 95%, lowest expected specificity (SP) to be 80%, confidence interval (W) for both sensitivity and specificity to be 5% and prevalence of neonatal jaundice, anemia and hypoxia to be 15%^2,3,46^ the minimal sample size required to achieve the targeted sensitivity and specificity were found to be 487 and 290 each respectively. Hence, the effective population size is 2331. However, we decided to include a much higher number (N = 4318) of subjects in our study to reach a more robust statistical outcome. Out of this, 3427 subjects were analyzed using our developed algorithm. The remaining 891 subjects were excluded on the basis of our proposed algorithm.

### Study design and subjects

The study included 4668 neonates with gestational age from 28 to 40 weeks. Among them, 70 subjects failed the recruitment criteria, of which 47 subjects had cannula on either of the hands and 9 patients had other complications (e.g., inaccessible thumbnail, uneven nail bed, or other physical problems with the thumb), and were thus excluded. Based on deteriorated blood samples (hemolysed blood samples, delayed blood processing, inadequate blood volume, and ambiguous blood information), 223 patients were further ruled out from the analysis. Hence, the effective population size of 4318 neonates was considered for the study. Comprehensive details of the subjects are provided in Table 2. Out of this, 3689 subjects were analyzed using our developed algorithm. The remaining 630 subjects were excluded on the basis of our proposed algorithm. Particulars about inclusion and exclusion criteria are described in Table 3. Out of the 3689 neonates, measurements from 1784 subjects were used for the training or calibration of the device. The rest of the 1935 neonatal subjects were selected for the validation of the instrument.

**Table 2:** Demographic details of the neonates participated in the study.

Description		Number of subjects
Neonates (N)		4318
Trans cutaneous measurement (n)		4318
Mode of delivery	Spontaneous vaginal	1295
	Assistive vaginal	1727
	Elective C-section	864
	Emergency C-section	432
Gestational age (weeks)	< 35	950
	35–37^6/7^	1252
	38–39^6/7^	1080
	40	172
	Unknown	864
Birth weight	Low birthweight (LBW)	600
	Very low birthweight (VLBW)	384
	Extremely low birthweight (ELBW)	179
Gender	Male	2706
	Female	1929
	Ambiguous	32
Race	Asian	3475
Feeding	Breast (%)	3022
	Formula (%)	431
	Both (%)	863
	Unknown (%)	2
Post-natal age	≤ 24 h	472
	24–47.9 h	579
	48–71.9 h	836
	≥ 72 h	3105
Disorders	Congenital heart disease	278
	Respiratory distress syndrome	398
	Pneumonia	123
	Rh incompatibility	143
	Birth asphyxia	509
	ABO incompatibility	362
	Others*	2505

*Other disorders include hypoglycemia, sepsis, infant of diabetic mother, jitteriness, premature rupture of the membranes (PROM), apnoea of prematurity, maternal varicella, intra uterine growth retarded (IUGR), hepatosplenomegaly, torch (+ ve, HSV, CMV), congenital rubella (IgM + ve), hypothyroid, osteogenesis imperfect, meningitis, Pierre Robin Syndrome and chrorioamnionitis.

**Table 3:** Inclusion and exclusion criteria for the study.

Inclusion criteria	Inclusion criteria comprised of all the neonates irrespective of the gestational age admitted to the Department of Paediatric Medicine, NRSMH with or without jaundice or anaemia or hypoxia and whose parents were willing to provide written informed consent after getting detailed information about the study
Exclusion criteria	Neonates having cannula in either of the hands Neonates who are extremely sick and from whom blood samples cannot be drawn Babies having major congenital malformation Neonates having lower peripheral circulation, etc
Jaundiced subjects	Neonates having bilirubin level above the Bhutani nomogram and is undergoing phototherapy
Anemic subjects	Neonates with hemoglobin level less than 11 gm/dL
Hypoxic subjects	Neonates with SpO_2_ less than 92%

It is worth mentioning that the recruitment of neonates was not consecutive as not all physicians practicing in the department were involved in the study. The neonates getting treatment under the physicians associated with the study were inducted. The appearance of possible selection bias was avoided following the approach described by Hammer et al.^47^. Random assignment of doctors (a general policy for the public hospitals in India), large time frame of the study (15 months), sufficiently large sample size, collection of data throughout 24 h window, and enough number of subjects in each subcategory (i.e., stratification of samples) helped in avoidance of the sampling bias.

### Quality assurance in data collection

Care was taken that a similar clinical protocol i.e., study, reference, and sample collection methods, and patient enrolment strategies were prospectively maintained throughout the experimental period. To avoid bias in measurements, particular care was taken to keep the technicians, clinicians, investigators, and data analysts at data collection sites blinded to the SAMIRA and the hematological data. Data of each neonate on pre-defined variables like the date, identification number, sex, gestational age, maternal history, whether having any risk factors, treatment details, etc. was collected from clinical charts on a tablet having required database with the in-built proforma by one laboratory technician hired for the study purpose. Blood collection, serum isolation, and measurements by SAMIRA were performed by trained nurses of the Department of Pediatric Medicine, NRSMH. They were responsible for uploading the SAMIRA readings to the database. The hematological parameters were measured by expert clinical biochemists at Central Laboratory, NRSMH who were completely unaware of the study. The TSB, Hb and arterial blood gas readings with proper identification numbers of the selected subjects were uploaded by another laboratory technician hired for the study purpose. The readings of both the methods (SAMIRA and the conventional) were matched based on the identification number by one research staff, to ensure complete blindness of the study. Complete blinding was maintained to keep the two sets of readings separate.

### Hematological measurement

For simultaneous measurement, about 2 mL of blood was collected for the conventional TSB, Hb and arterial blood gas measurement test within 30 min of the data collected from SAMIRA.

The TSB of the subjects was quantitatively determined by the 2,5-dichlorophenyldiazonium tetrafuoroborate (DPD) diazo method described by Jendrassik and Groff^48^, using the commercially available test kit (Autospan Liquid Gold, Span Diagnostics, India) within 1 h of blood collection in the Central Laboratory, NRSMH. For the test, serum was first isolated from the collected blood and then examined with the test kit. To prevent the photoreduction of bilirubin the serum samples were carefully kept in the dark at 4 °C before analysis.

For hemoglobin and arterial blood gas measurement the collected blood samples were subjected to an automated hematology analyzer (Sysmex KX-21)^49^ for complete blood count (CBC) analysis and GEM premier 3000 system^50^ (Instrumentation Laboratory. Bedford, MA), respectively.

All the guidelines provided by the National Accreditation Board for Testing and Calibration Laboratories (NABL)^51^ were followed to maintain the accuracy and precision of the techniques. The coefficient of variation for the hospital laboratory was targeted for < 6%. During the study period, each of the actual variance values, assessed every 3 months, ranged from 3 to 5%.

### Statistical analysis

Analysis of the data was done using descriptive statistical analysis, simple linear regression analysis, and the Bland & Altman method^52–55^. For the correlation between the values obtained from the device and the gold standard, linear regression and Bland Altman was used. The mean (n) of the measurements was calculate according to the formula:

8 $$n= \frac{Sum\, of\, the\, data\, points}{Number\, of\, data\, points}$$

The Standard deviation between the successive measurements was measured using the formula

9 $$SD= \sqrt{\frac{{\sum }_{i=0}^{N}\left({x}_{i}-\mu \right)}{N}}$$

where, N refers to the number of experimental outcomes, µ is the mean of the individual outcomes and *x*_*i*_ is each outcome of the experiment. GraphPad Prism 5.0 (GraphPad Sofware, USA) and SigmaPlot 12.5 (Systat Sofware, USA) were utilized for the analysis of the data.

### Ethical considerations

For the present work, all necessary ethical permissions were taken from the Institutional Medical Ethics Committee, NRSMH, Kolkata (Ref. No.—No/NMC/439, dated January 27, 2020). All studies involving human subjects were performed following the Declaration of Helsinki^56^ and guidelines provided by the Indian Council for Medical Research (ICMR), Govt. of India. Written informed consent was obtained from parents or legal guardians who agreed to participate in the study after understanding the details of the study and its consequences. All data and information about the subjects were anonymized, kept confidential and used only for this study.

## Results

### Calibration of the instrument

In order to acquire the optimum condition for data acquisition, we performed data analysis using the self-devised algorithm in 3 different partitions on the calibration dataset. The dataset was divided into 3 partitions depending on the amount of scattered data points from the cumulative fitted curve. The three partitions are 90% (when 90% of the data points are within the range of ± 0.02); 95% (when 95% of the data points are within the range of ± 0.02) and 98% (when 98% of the data points are within the range of ± 0.02).

The correlation plot of the instrument at the three partitions with the three blood parameters calculated from the standard biochemical test on 1784 neonatal subjects shows three patterns of dependency on the blood parameters (Fig. 3g–o). Figure 3g–i shows the second order polynomial dependency of the instrument index values with TSB levels for all the three partitions. The Adjusted r^2^ was found to be as follows: when 90% of the data points are within the range of ± 0.02, 0.77. When 95% of the data points are within the range of ± 0.02, the adjusted r^2^ is 0.96 and when 98% of the data points are within the range of ± 0.02, the adjusted r^2^ is 0.97. The instrument index values maintain a linear relationship with the Hb values obtained from blood tests for all the partitions (Fig. 3j–l). The Pearson’s correlation coefficient, r was found to be 0.969 (slope = 1.7622; intercept = 2.862) when 90% of the data points are within the range of ± 0.02. When 95% of the data points are within the range of ± 0.02, correlation coefficient, r was found to be 0.972 (slope = 1.911; intercept = 1.75) and when 98% of the data points are within the range of ± 0.02, the correlation coefficient, r was 0.9745 (slope = 1.8; intercept = 2.91). Moreover, a fourth order polynomial dependency of the instrument index values was obtained with the arterial blood gas for all the three partitions of the datasets (Fig. 3m–o). The Adjusted r^2^ was found to be as follows: when 90% of the data points are within the range of ± 0.1. is 0.953; When 95% of the data points are within the range of ± 0.05, the adjusted r^2^ is 0.974 and when 98% of the data points are within the range of ± 0.02, the adjusted r^2^ is 0.989.

### Validation of the instrument

**Figure 4:**
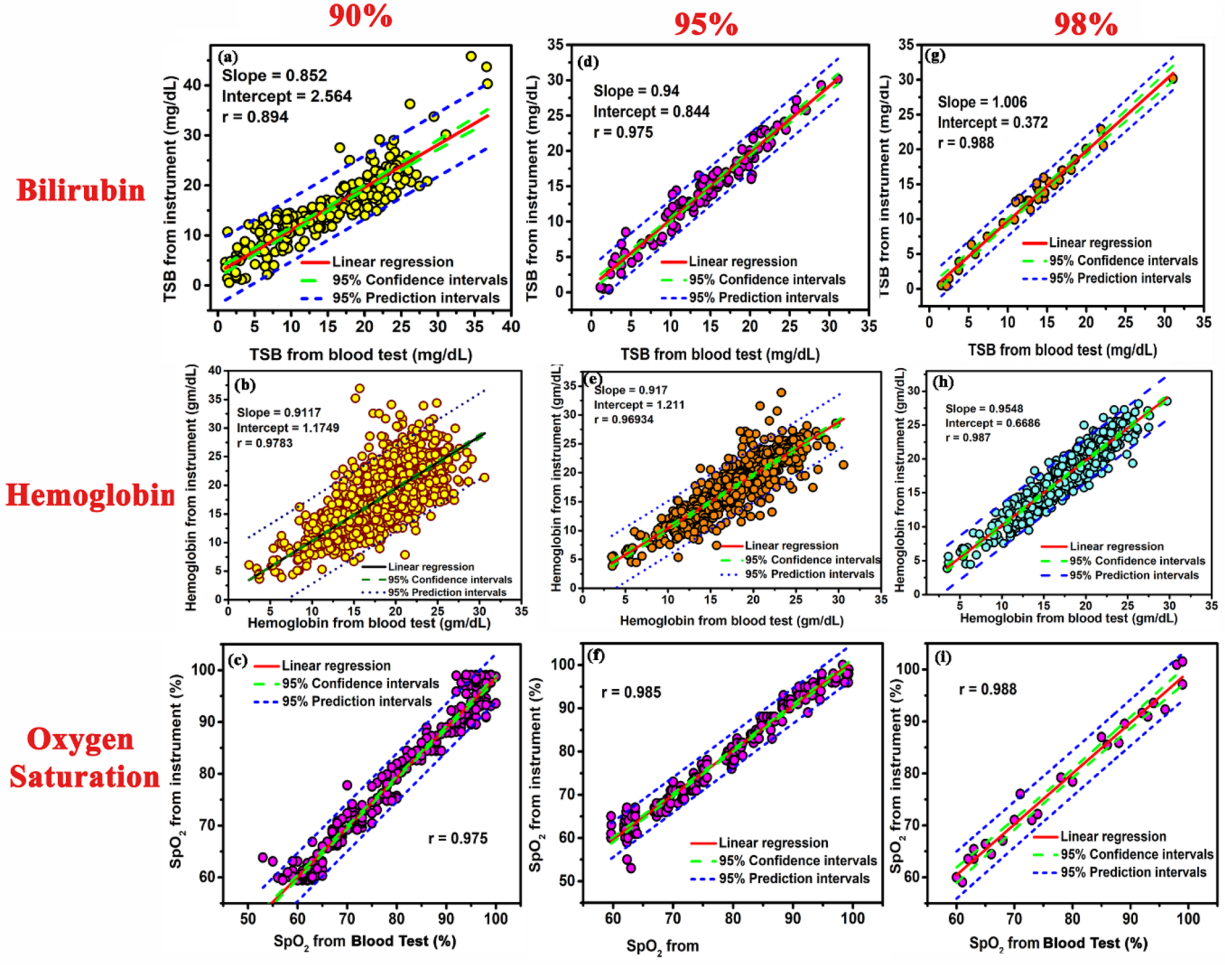
Validation of the instrument in different partitions of the dataset. Linear regression plot of the developed device verses TSB on 230 neonates, when (**a**) 90% of the acquired data points are scattered within ± 0.02% from the cumulative fitted curve in the residual plot. (**d**) 95% of the acquired data points are scattered within ± 0.02% from the cumulative fitted curve in the residual plot. (**g**) 98% of the acquired data points are scattered within ± 0.02% from the cumulative fitted curve in the residual plot. Linear regression plot of the developed device verses Hb on 1073 neonates, when (**b**) 90% of the acquired data points are scattered within ± 0.02% from the cumulative fitted curve in the residual plot. (**e**) 95% of the acquired data points are scattered within ± 0.02% from the cumulative fitted curve in the residual plot. (**h**) 98% of the acquired data points are scattered within ± 0.02% from the cumulative fitted curve in the residual plot. Linear regression plot of the developed device verses SpO_2_ on 340 neonates, when (**c**) 90% of the acquired data points are scattered within ± 0.02% from the cumulative fitted curve in the residual plot. (**f**) 95% of the acquired data points are scattered within ± 0.02% from the cumulative fitted curve in the residual plot. (**i**) 98% of the acquired data points are scattered within ± 0.02% from the cumulative fitted curve in the residual plot.

A total number of 1935 neonates were selected for validation of the instrument (Fig. 4). The instrument was validated in 409 subjects suffering from neonatal jaundice. 65 anemic subjects, and 223 subjects suffering from hypoxia. The remaining 1238 subjects were taken as control population for the purpose of validation of the device. All the datasets were divided into three partitions and the analyzed using the developed algorithm. Linear regression and Bland–Altman analysis was performed to evaluate the correlation between the obtained data produced by the instrument with the gold standard clinical laboratory tests.

**Figure 5:**
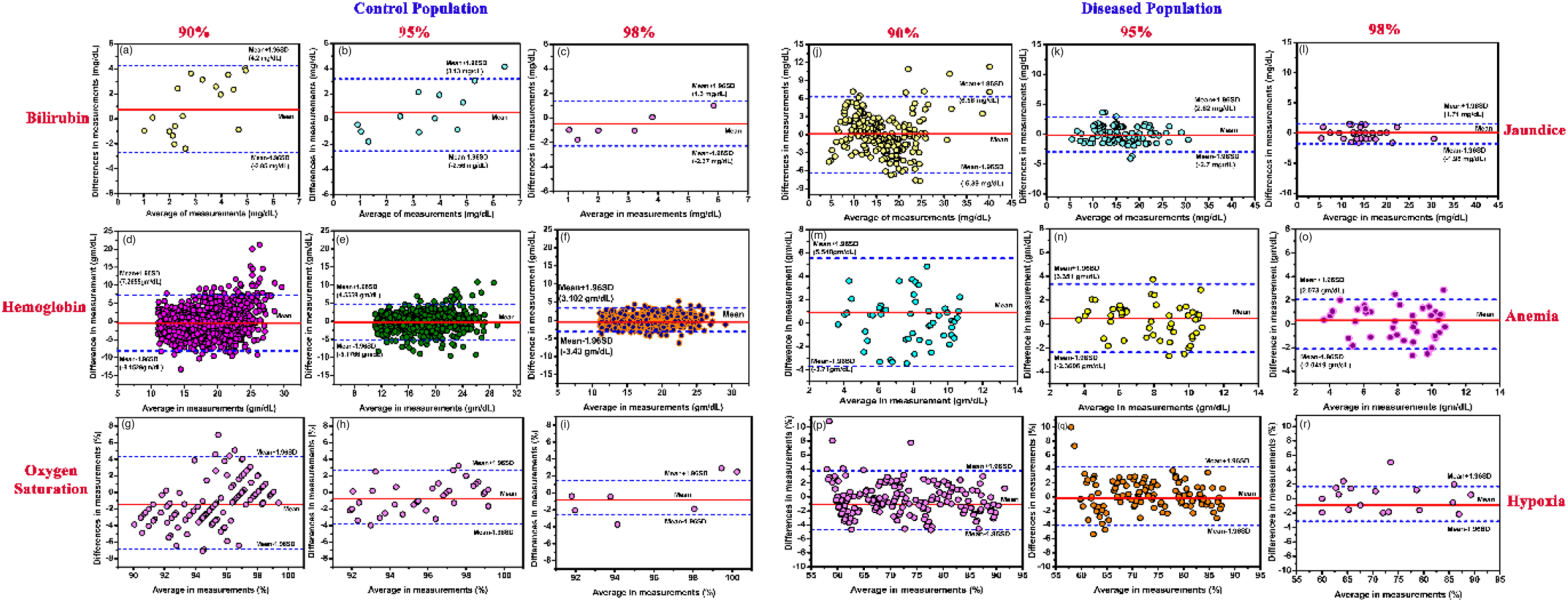
Relationship between the developed device and the blood parameters obtained from blood sampling in normal neonates (Control population) and diseased neonates. Bland–Altman plots (mean and 95% limits of agreement) between the developed device and TSB values in control and jaundiced subjects respectively when, (**a**,**j**) 90% of the acquired data points are scattered within ± 0.02% from the cumulative fitted curve in the residual plot. (**b**,**k**) 95% of the acquired data points are scattered within ± 0.02% from the cumulative fitted curve in the residual plot. (**c**,**l**) 98% of the acquired data points are scattered within ± 0.02% from the cumulative fitted curve in the residual plot. Bland–Altman plots (mean and 95% limits of agreement) between the developed device and Hb values in control and anemic subjects respectively when, (**d**,**m**) 90% of the acquired data points are scattered within ± 0.02% from the cumulative fitted curve in the residual plot. (**e**,**n**) 95% of the acquired data points are scattered within ± 0.02% from the cumulative fitted curve in the residual plot. (**f**), (**o**) 98% of the acquired data points are scattered within ± 0.02% from the cumulative fitted curve in the residual plot. Bland–Altman plots (mean and 95% limits of agreement) between the developed device and ABG values in control and hypoxic subjects respectively when, (**g**,**p**) 90% of the acquired data points are scattered within ± 0.02% from the cumulative fitted curve in the residual plot. (**h**,**q**) 95% of the acquired data points are scattered within ± 0.02% from the cumulative fitted curve in the residual plot. (**i**,**r**) 98% of the acquired data points are scattered within ± 0.02% from the cumulative fitted curve in the residual plot.

Linear regression analysis between the values obtained from the instrument at the three partitions with the blood tests show a more correlation when 98% of the data points are within the range of ± 0.02 (for TSB estimation, r = 0.988; slope = 1.006; intercept = 0.372; for Hb estimation, r = 0.987; slope = 0.954; intercept = 0.668; for estimation of oxygen saturation, r = 0.988slope = 0.977 ; intercept = 1.775 , Fig. 4g–i) in comparison when 95% of the data points are within the range of ± 0.02 (for TSB estimation, r = 0.975; slope = 0.94; intercept = 0.844; for Hb estimation, r = 0.969 slope = 0.917; intercept = 1.211; for estimation of oxygen saturation, r = 0.985; slope = 1.029; intercept = 2.098, Fig. 4d–f). On the contrary, when 90% of the data points are within the range of ± 0.02 the correlation was reduced (for TSB estimation, r = 0.894; slope = 0.852; intercept = 2.564; for Hb estimation, r = 0.9783; slope = 0.911; intercept = 1.1749; for estimation of oxygen saturation, r = 0.975 slope = 0.961; intercept = 2.375, Fig. 4a–c). Bland–Altman analysis (Fig. 5) also corroborated the highest correlation between the when 98% of the data points are within the range of ± 0.02 and the obtained blood parameter values of TSB, Hb, and arterial blood gas for both normal and diseased neonatal subjects in comparison to the other two partitions. The statistical parameters when 98% of the data points are scattered within a range of ± 0.02 are as follows: with the TSB values obtained from the biochemical test, (bias for normal = − 0.491 mg/dL, jaundiced subjects = 0.099 mg/dL; 95% limits of agreement for normal = − 2.37 mg/dL to 1.3 mg/dL and jaundiced subjects = − 1.98 mg/dL to 1.71 mg/dL, Fig. 5c,l); with Hb values obtained from the blood tests (bias for normal; − 0.08 gm/dL, anemic; 0.19 gm/dL; 95% limits of agreement for normal; − 3.43 gm/dL to 3.10 gm/dL, anemic; − 2.04 gm/dL to 2.07 gm/dL and − 0.93 gm/dL to 1.23 gm/dL, Fig. 5f,o) and with the arterial gas values (bias for normal = − 0.56% and hypoxic = − 1.05%; 95% limits of agreement for normal = − 2.38% to 1.38% and hypoxic = − 3.45% to 1.6%, Fig. 5i,r). The statistical parameters when 95% of the data points are within the range of ± 0.02 are as follows: with the TSB values obtained from the biochemical test, (bias for normal = 0.25 mg/dL, jaundiced subjects = − 0.11 mg/dL; 95% limits of agreement for Normal = − 2.56 mg/d to 3.13 mg/dL, Jaundice subjects = − 2.7 mg/dL to 2.62 mg/dL Fig. 5b,k); with Hb values obtained from the blood tests (bias for normal = − 0.3104 gm/dL anemic = 0.4952 gm/dL; 95% limits of agreement for Normal = − 5.17 gm/dL to 4.55 gm/dL, Anemic = − 2.36 gm/dL to 3.35 gm/dL Fig. 5e,n) and with the arterial gas values (bias for Normal = 1.13%, Hypoxic = − 0.23%; 95% limits of agreement for Normal = − 4.49% to 2.64%, Hypoxic = − 4.16% to 4.32% Fig. 5h,q). However, when the 90% of the data points are within the range of ± 0.02, the statistical parameters are as follows: with the TSB values obtained from the biochemical test, (bias for normal = 0.37 mg/dL, jaundiced subjects = 0.153 mg/dL; 95% limits of agreement for normal = − 2.85 mg/dL to 4.2 mg/dL and jaundiced subjects = − 6.39 mg/dL to 6.56 mg/dL, Fig. 5a,j); with Hb values obtained from the blood tests (bias for Normal = − 0.43 gm/dL, Anemic = 0.91 gm/dL; 95% limits of agreement for Normal = − 8.14 gm/dL to 7.26 gm/dL, Anemic = − 3.71 gm/dL to 5.54 gm/dL, Fig. 5d,m) and with the arterial gas values (bias for Normal = − 1.05%, Hypoxic = − 1.4%; 95% limits of agreement for Normal = − 6.56% to 4.17%, Hypoxic = − 4.78% to 3.83% Fig. 5g,p). 95% limits of agreement means that 95% of the differences are assumed to lie within these limits and how far apart the measurements obtained using the two methods are likely to be for most individuals. Bias refers to the difference between the expected value obtained from the device and the true value of the parameter being obtained from the haematological tests. Detailed results of comprehensive statistical analysis are presented in Table 4.

**Table 4:** Statistical parameters defining the correlation between the developed device and the blood test in a diseased population.

Category	Statistical test	Parameters	Values
Anemic subjects (Hb < 11 gm/dL)	Bland Altman analysis	Bias (95% CI)	0.19 gm/dL
		Standard deviation	1.401
		Limits of agreement	−2.0419 gm/dL to 2.073 gm/dL
Jaundiced subjects	Bland Altman analysis	Bias (95% CI)	0.099 mg/dL
		Standard deviation	0.9451 mg/dL
		Limits of agreement	− 1.98 mg/dL to 1.71 mg/dL
Subjects with heart diseases	Bland Altman analysis	Bias (95% CI)	− 1.05%
		Standard deviation	1.93
		Limits of agreement	− 3.45 to 1.6

### Repeatability

**Figure 6:**
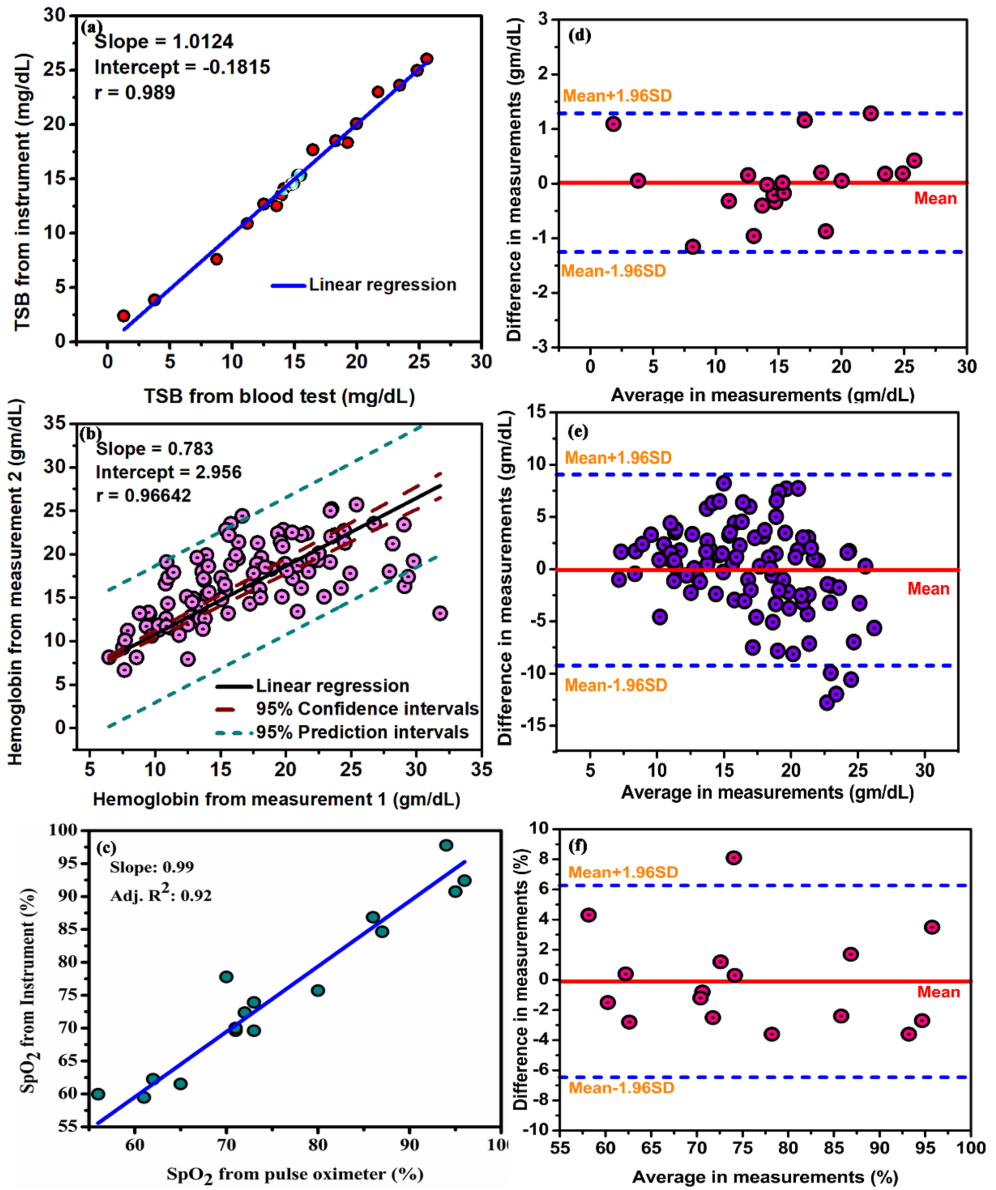
Repeatability of the data acquired using the developed device. Linear Regression analysis for five successive values of (**a**) Bilirubin (**b**) Hemoglobin (**c**) SpO_2_ measurement on the same subject by the same observer. Bland–Altman analysis for five successive values of (**d**) Bilirubin (**e**) Hemoglobin (**f**) SpO_2_ measurement on the same subject by the same observer.

In order to check the repeatability of the device, measurement was taken from the neonate’s nail bed 5 times by the same observer. The repeatability of the device to parameterize the hemoglobin, bilirubin and oxygen saturation values was performed in 135 neonates (Fig. 6).

We found a standard deviation of 3.2 mg/dL between back-to-back measurements in the same subject by the same observer in the estimation of bilirubin. For the estimation of hemoglobin and oxygen saturation, the standard deviation values were found to be 4.6 gm/dL and 3.2% respectively. The calculated SD and mean were almost the same in both the measurements, for all the three blood parameters. The linear regression analysis between the two measurements further confirmed the accuracy of the two measurements. For the repeatability analysis, the correlation between the measurements are as follows: for TSB measurement, (r = 0.989; slope = 1.0124; intercept = 0.1815, P < 0.001), for the estimation of Hb (r = 0.96642; slope = 0.783; intercept = 2.95, P < 0.001), and for the measurement of SpO_2_ (r = 0.963; slope = 0.99, P < 0.001). Bland Altman analysis was performed to confirm the correlation between successive measurements; for TSB measurement (bias = 0.183 mg/dL, 95% limits of agreement = − 1.25 to 1.287 mg/dL) for Hb estimation (bias = − 0.85gm/dL 95% limits of agreement = − 9.2 gm/dL to 9.05 gm/dL) for estimation of oxygen saturation (bias = − 1.01% 95% limits of agreement = − 6.46% to 6.25%).

## Discussion

The conventional non-invasive trans cutaneous methods are yet to replace the invasive method of blood sampling due to certain shortcomings of the transcutaneous devices. The TcB (trans cutaneous bilirubin) measured by these non-invasive devices consists of a major contribution from the extravascular bilirubin, which is a completely a different physiological parameter in comparison to the TSB. The unpredictable process that regulated the dynamics of bilirubin in the extravascular space makes a one to one comparison of TSB and TcB impossible^26,27^. Confinement of the measurement volume only to the intravascular space could help in overcoming the problem^27^. Whereas, the Hb and the SpO_2_ values acquired by the available transcutaneous hemoglobin meters and pulse oximeters have a significant positive bias^31,57^. This in turn, causes improper management particularly to the diseased population that tend to get their Hb and blood saturation values over estimated. The SAMIRA device is based on such spectroscopy based approach where the information is collected from the vascular bed underneath the nail plate^55^. Thus, we hypothesize that SAMIRA will be able to overcome the limitations of the conventional trans cutaneous devices for the simultaneous measurement of three blood parameters from a single optical spectrum.

Our results suggests that the bilirubin, hemoglobin, and SpO_2_ values obtained from SAMIRA has a positive linear correlation with all the three blood parameters (for Hb measurement, r = 0.96; for bilirubin measurement, r = 0.98; for SpO_2_ measurement, r = 0.98). Using the self-devised algorithm and multivariate regression analysis, we observed that when 95% of the acquired data points are within the range of ± 0.02, the optimum condition for acquiring reliable data from the neonates was achieved. Furthermore, the demographic analysis (Table 2) showed that postnatal age, exclusive breastfeeding, gestational age or any other risk factor are not associated with any variation in the performance of the device. No bias of birth weight and/or gestational age was found to observe on the non-invasive blood parameter measurements in the intensive study on 4318 neonatal subjects. Moreover, as the study was conducted on a subgroup of Indian population, the interference of dark skin colour (or variation within the Indian subpopulation) can also be ruled out, as Indian population consists of mixed races with varied skin tones. Skin tone was not found to be a confounding factor for the estimation of Hb, TSB or SpO_2_. However, the device slightly overestimated the bilirubin values by 5 mg/dL when the TSB exceeded 15 mg/dL. Otherwise, it marginally overestimated the bilirubin values by 2 mg/dL. Although this may result in unnecessarily prolonged hospitalization, it eliminates the chances of serious clinical errors like mismanagement of a diseased infant and hence decreases infant morbidity and mortality.

The correlation coefficient between SAMIRA and the arterial blood gas measurements was found to be 0.98, which is more than the other pulse oximeter devices where the correlation coefficient is of the order of 0.8 to 0.85^58^. Furthermore, the correlation with SpO_2_ < 70%, was found to be 0.98, which was much higher than the other non-invasive pulse oximeters with a correlation coefficient of 0.8–0.85^30^, which tends to overestimate the SpO_2_values below 70%. The correlation coefficient between the developed device and the TSB values was found to be 0.88, which was better than the correlation shown other non-invasive devices which was of the order of 0.7–0.8^59,60^. It has to be noted that these studies were conducted on white population in which the conventional TcB meters generally show good efficacy. Several studies have reported that TcB meters overestimate bilirubin values in dark skin populations like Hispanic, Asian, African, etc.^24,28,61^.In our study values of overestimation by ≥ 2 mg/dL, ≥ 3 mg/dL and ≥ 4 mg/dL were found to be 25%, 4.2% and 0.9% respectively. To the best of our knowledge, no TcB device has been developed to address bilirubin overestimation in the black population, and a low-cost, non-invasive, point of care device for these ethnic groups holds promise for low and middle income countries^62^. The correlation of the device in the anemic region (Hb < 11 gm/dl) was found to be 0.99, which was highly comparable with the commercially available devices with a correlation coefficient of 0.95^22,23^. It has to be noted that these available instruments estimated the hemoglobin values in adults. Due to the different architecture and thickness of the skin, adult hemoglobin meters are not a suitable comparison to the neonatal trans cutaneous hemoglobin meters. The Bland–Altman analysis confirmed that SAMIRA was highly sensitive in the anemic region, which suggests that even the mildest form of anemia can be detected by the device.

In some studies, it has been shown that pulse oximetry usually overestimates the SpO_2_ values less than 90% in cyanotic children^31,63,64^ However, the correlation between the developed device with the SpO_2_ values less than 90% is 0.99. The limits of agreement is between − 20.9% to 10.3%^31^ for the other pulse oximeters which shows, that each point suffers from huge standard deviation. Whereas, the limits of agreement are within − 4.3% to 5.3% of our developed device, which ensures lesser fluctuation of the data points.

This is one of the few studies that extensively assessed the performance of a non-invasive device in neonates suffering either from hyperbilirubinemia or anemia or hypoxia. We think that the strength of the study lies in the prospective design, a large number of sample in each category, the intra-racial variation in the dark skin color, the parallel measurement of three blood parameters (bilirubin, hemoglobin, arterial blood gas) by two methods (SAMIRA and the biochemical tests), and the collection of the blood for the routine biochemical tests by regular nurses for clinical use rather than specifically for the study purpose in which conditions might be optimized. The conventional measurements were performed by the experienced clinical biochemists and laboratory technicians of a tertiary care hospital, reducing the possibility of operator error. Overall, we believe that our results provide a robust estimation of the accuracy of non-invasive hemoglobin, bilirubin and SpO_2_ measurement by a new device and the sources of error are applicable to routine clinical settings.

Our study had few limitations over the other transcutaneous devices. The available transcutaneous instruments did not perform well on subjects having a bilirubin report of more than 15 mg/dL.^65,66^. In subjects where the bilirubin crossed 15 mg/dL, it was associated with severe complications like exchange transfusion and was avoided for complications. Another confounding factor of the study was that measurements on neonates with less than 500 g of body weight was not considered for the study due to their added complications. Studying the population having high TSB values and also associated with the risk factors for hyperbilirubinemia, anemia and hypoxia, and manipulation of the intensity of illumination can further increased the accuracy of the device. Lastly, the results obtained from the non-invasive measurements (SAMIRA) were used for clinical management. A real-time clinical management using these readings has helped to assess the actual accuracy of this device to reduce painful blood sampling in day-to day clinical practice.

## Conclusion

The new non-invasive non-contact device (SAMIRA) can accurately measure hemoglobin, bilirubin and SpO_2_ levels simultaneously from a single optical spectrum. It could also measure TSB > 20 mg/dL, which was eliminated by the modification in the illumination intensity of the source. Interestingly, in this study we also found that the device gave accurate results to predict the onset of heart diseases in neonates by measuring their blood saturation levels. This will help clinicians to better monitor the neonates and reduce the frequency of blood sampling. Moreover, the device collects the data from the neonate’s nail bed which contains less melanin interference irrespective of the skin tone of the neonate. Overall, our results suggest that our device can be used in hospital settings for the accurate measurement of hemoglobin, bilirubin and SpO_2_ for the screening of hyperbilirubinemia, anemia and heart diseases in neonates.

## Data Availability

Data that support the findings of this study are available with the corresponding author upon reasonable request. Figures 1, 2, 3, 4, 5 have associated raw data which is available to the corresponding author on request.

## Code availability

Code for the algorithm is available with the corresponding author and can be shared with the readers on request.
